# ‘Antibiotic footprint’ as a communication tool to aid reduction of antibiotic consumption—authors’ response

**DOI:** 10.1093/jac/dkz533

**Published:** 2019-12-22

**Authors:** Direk Limmathurotsakul, Jonathan A T Sandoe, David C Barrett, Michael Corley, Li Yang Hsu, Marc Mendelson, Peter Collignon, Ramanan Laxminarayan, Sharon J Peacock, Philip Howard

**Affiliations:** 1 Mahidol-Oxford Tropical Medicine Research Unit, Faculty of Tropical Medicine, Mahidol University, Bangkok, 10400, Thailand; 2 Department of Tropical Hygiene, Faculty of Tropical Medicine, Mahidol University, Bangkok, 10400, Thailand; 3 Centre for Tropical Medicine and Global Health, University of Oxford, Oxford OX3 7FZ, UK; 4 University of Leeds/Leeds Teaching Hospitals NHS Trust, Leeds LS1 3EX, UK; 5 British Society of Antimicrobial Chemotherapy, Birmingham B1 3NJ, UK; 6 Bristol Veterinary School, University of Bristol, Bristol BS40 5DU, UK; 7 Saw Swee Hock School of Public Health, National University of Singapore and National University Health System, 12 Science Drive 2, Singapore 117649, Singapore; 8 National Centre for Infectious Diseases, Moulmein Road, Singapore 308433, Singapore; 9 Division of Infectious Diseases & HIV Medicine, Department of Medicine, University of Cape Town, Cape Town, 7925, South Africa; 10 International Society for Infectious Diseases, Brookline, MA 02446, USA; 11 Infectious Diseases and Microbiology, Canberra Hospital, Canberra, 2605, Australia; 12 Medical School, Australian National University, Acton, 2606, Australia; 13 Center for Disease Dynamics, Economics & Policy, New Delhi, 110024, India; 14 Princeton Environmental Institute, Princeton, NJ 08544, USA; 15 Department of Medicine, University of Cambridge, Cambridge CB2 0QQ, UK

Sir,

We thank Gould *et al*.^1^ for their comments on our article on the antibiotic footprint, which we proposed as a simple communication to aid public understanding of antibiotic consumption.^2^ We fully agree that the antibiotic footprint could also be used as a tool to support interventions to reduce the overuse of antibiotics.^1^ We understand the possibility of presenting the antibiotic footprint together with thresholds of antibiotic consumption in different hospitals and communities, defined as number of treatment courses, or DDDs per unit of hospital activity or community population, as presented by Gould *et al*.,^1^ additionally recognizing that a similar approach could be taken on livestock farms and in other veterinary and one-health contexts. However, complete data on antibiotic usage as proposed^1^ are rarely available in low- and middle-income countries (LMICs) and further studies evaluating whether thresholds of antibiotic consumption could be defined for each LMIC are needed. The information on complete antibiotic use and new evidence arising from such studies would certainly allow the communication strategy to be adjusted and improved over time.^2^ Furthermore, we strongly agree with the recent report, ‘Reframing Resistance’, published by the Wellcome Trust,^3^ which proposed that communication messages to the public need to be tested and that including the issue of antibiotic overuse in both humans and animals in the right way helps make the issue feel tractable. We foresee that the concept of the antibiotic footprint could be expanded to online individual calculators^2^ and that local thresholds of antibiotic consumption could help in guiding local appropriate antibiotic use in the community.^1^ However, further studies to evaluate the use of the antibiotic footprint and antibiotic consumption thresholds to communicate with communities are needed, particularly in different languages and contexts.

## Transparency declarations

None to declare.
